# The Nature and Treatment of Dropsy Considered, Especially in Reference to the Diseases of the Internal Organs of the Body Which Most Commonly Produce It. Parts I. And II. Anasarca and Ascites, &c.

**Published:** 1837-01-01

**Authors:** 


### Medico-Chirurgical Review,

### No. LI.

**[No. 11 of a Decennial Series.]**

OCTOBER 1, 1836, TO JANUARY 1, 1837.

The Nature and Treatment of Dropsy; considered especially in reference to the Diseases of the Internal Organs of the Body which most commonly produce it. Parts 1 and 2—Anasarca and Ascites. To which is added an Appendix, containing a Translation of the Work of Dr. Geromini, on Dropsy, from the original Italian. By *Edward J. Seymour*, M.D. Physician to St. George's Hospital, Consulting Physician to the Seaman's Hospital, and one of the Physicians in Ordinary to H.R.H. the Duke of Sussex. Octavo, pp. 218. London, 1837.

THOSE who have the pleasure of an acquaintance with Dr. Seymour know that he is a physician of experience, of observation, and of that practical turn of mind, which ensures his not wasting either observation or experience on subtle and useless hypotheses. These considerations might lead us to anticipate, that a work on Dropsy from his hands would be one of an interesting, because a practical character, and such anticipations have been amply realized by a careful perusal of the work.

The frequency of those organic lesions which are attended with one form or other of dropsy—the necessity of discriminating the effect from the cause, and the varieties of that effect from each other—the prevalence of the error, which originated in the want of a knowledge of morbid anatomy, the error of commonly erecting dropsy into a substantive disease—and the general deficiency of accurate ideas of the nature of the disorder, and of definitive principles of treatment, induce us to seize the present opportunity of laying a full analysis of this work before our readers. We are mistaken if they do not find it of considerable utility.

The first part is devoted to the investigation of anasarca, or dropsy of the skin. It contains five chapters. The first chapter is on anasarca in general—the second, on anasarca from rheumatic disease of the heart—the third on anasarca from enlargement of the heart—the fourth, on anasarca occurring independently of organic disease of an internal organ—the fifth, on anasarca from disease of the kidneys.

The second part is appropriated to ascites, or dropsy of the abdomen. It treats of it as it arises from contraction of the liver, or from diseases of the viscera supervening upon ague. The last form of ascites discussed, is that which depends on disease of the peritoneum.

Of the appendix we need say nothing at present, but proceed to analyse the observations of Dr. Seymour on the subjects we have enumerated.

### ANASARCA, OR DROPSY OF THE SKIN.

Dr. Seymour observes, with justice, that probably there is no disease on which pathological anatomy has thrown more light than upon dropsy. It is, indeed, one of those complaints to which we may triumphantly point, when asked what morbid anatomy has done. Dropsy is no longer, by scientific physicians, regarded as, in the majority of cases, a substantive disease, but rather as a symptom of organic alteration of one or more of the viscera. In most instances, it arises from the return of blood to the right side of the heart being obstructed, and from the impaired functions of that organ being relieved by the secretion of large quantities of fluid; the effusion being partial where the obstruction is partial, but where the obstruction is in the heart itself, sooner or later extending through the whole cellular texture.

To exhibit the several efficient causes of anasarca, our author graphically describes four cases—suppositious in form, but in reality the representatives of large classes of facts.

1. "A patient, between twenty and thirty, or younger, applies for relief, having difficulty of breathing, increased on ascending a stair, or on rapid motion, with constant palpitation of the heart: he will relate that he has suffered from this palpitation for several years, on any hurry, but that latterly it has become constant; that he lies down in bed with difficulty,* and that latterly his ankles and legs have begun to swell; he has constant thirst; the urine is high-coloured; there is short dry cough, though he can inspire freely; his nights are restless; and when he wakes, his eyelids and cheeks are swollen: the pulse may be either quick and weak, or full and soft—rarely hard; the urine, on examination, does not coagulate by the admixture of acids, or by heat." 3.

On close inquiry, we find that at some former period (it may be remote) the patient has suffered from acute rheumatism, the articular inflammation having shewn a disposition to shift its seat, and having left more or less of palpitation behind it. The swelling has probably recently appeared. There is usually a bruit in the cardiac sounds, and much action is perceived in the vessels of the neck.

The cause of the disease is enlargement of the heart, from adhesions of the pericardium, and the dropsy is the effect of the difficulty of the circulation.

2. A man, generally under fifty years of age, is received into a hospital. He is enormously swollen: the face, hands, legs, and integuments of the abdomen hard, but retaining the impression of the finger; there is considerable difficulty of breathing, and hard dry cough; sometimes a little expectoration, frothy and tinged with blood; the urine is scanty, and does not

---

* "This symptom is not constant."

coagulate by the usual methods; the pulse is hard, sometimes full; the heart is felt beating over a large surface; the tongue is often foul. Such symptoms, Dr. Seymour assures us, depend on hypertrophy and dilatation of the heart, from hard living, and especially from porter drinking.

3. A woman, between 40 and 50, also applies for relief, with inordinate swelling of the whole body; but here the surface is not only distended and white, but the impression of the finger remains at considerable depth in the integuments: the pulse is quick and feeble, and even where there is volume it is easily compressible; but it is irregular, and there often is a grating sensation communicated to the finger. The urine is small in quantity, not very high coloured, but incoagulable by heat or acid. There is great difficulty of breathing, and often cough, with much mucous expectoration. Though the infiltration of the integuments of the abdomen at first leads to the idea of the existence of ascites, that is not really present. The case, according to our author is one of enlargement of the cavities, rather than of the muscular structure of the heart—attended, perhaps, with deposition of bone in the valves, and dilatation of the root of the aorta, with atheromatous deposits.

In all these cases, there is a greater or a less degree of congestion or condensation of the lungs.

4. A patient, probably young, applies with swelling of the lower extremities and of the face; the pulse is not weak; there is pain in the back, sometimes in either hypochondrium, and headache; no enlargement of the heart is perceptible; no violent palpitation; no movement over a large surface; thirst; no dyspnœa; the urine is abundant and pale coloured, but coagulates on the application of acids, or of heat. The dropsy in this case is the consequence of disease of the kidneys.

For the sake of distinctness, specimens of uncomplicated dropsy, arising from disease of a single organ have been separately presented. But not unfrequently more than one organ is altered, and, the causes being complicated, so are the effects.

The first chapter, at which we now arrive, is occupied with some general considerations on the causes of anasarca. The most frequent are—1, disease of the heart; 2, disease of the heart and lungs; 3, disease of the kidneys; 4, the sudden application of cold to the surface of the body; 5, eruptive fevers.

The simplest form of dropsy of the cellular membrane, is the swelling of a limb, or the extremity of a limb, without redness or heat, the part retaining, in a greater or less degree, the print of the finger impressed upon it, and the skin being white and shining: this is termed œdema. This may depend either on pressure on the veins above the swelling, or on obstruction of the cavity of the vein itself. An ovarian tumor, for example, pressing on the iliac vein occasions œdema of the lower extremity of the same side. Or, phlebitis ending in the deposition of lymph within the vein, and choking it, will also give rise to œdema of the limb below it. In cases of malignant growths, it is not uncommon to see the large veins in the vicinity loaded with the morbid matter, and to find œdematous swelling in consequence.

"Wherever great obstruction exists to the return of blood to the right side of the heart, it would be fair to conjecture, from what we see in œdema in a single limb, that the whole venous circulation would be congested, and hence the whole capillary arterial system would secrete fluid, as a relief to the obstruction; and if this did not occur, the balance of the circulation would be in so disturbed a state as to cause rupture of vessels in some vital structure; and this we find to be the case: the capillary arteries pour out fluid into the cellular membrane, of greater or less quantity, according as the obstruction is considerable, and of greater or less consistence as the patient's vital powers are greater or less, or the obstruction more or less sudden.

The œdema, in the first case, becomes now termed anasarca, or dropsy of the skin; the swelling is generally more in the legs, from their depending position, but the eyelids, cheeks, and integuments of the abdomen, are often greatly swollen, especially after sleep in a recumbent position. If the swelling pitted deeply on pressure, the skin being very white, and the forces of the patient broken, this condition was called by the ancients Leucophlegmasia; where the swelling was more tense and resistent, and strength less impaired, they employed the name Anasarca. As these are shades of the same disease only, I shall, for simplicity's sake, employ the latter term, as applicable to the whole disease." 11.

Anasarca, like œdema, is most commonly a symptom only, and usually it is a symptom of disease of the heart. The heart may be dilated, with or without hypertrophy. Dr. S. observes that the pulse is then increased in frequency, and sometimes but not always in force, and the heart is felt beating over a large surface. We regret that these observations are not more precise, for certainly, with the aid of auscultation, the diagnosis between dilatation with and dilatation without hypertrophy is very far from difficult or uncertain. In anasarca from enlargement of the heart, at an advanced period of life, our author assures us, that the valves are also frequently diseased, and he adds some diagnostic symptoms of the set of valves affected. If, he observes, the semilunar valves be the structure principally altered, the pulse at the wrist is smaller, while the heart beats turbulently and over a large surface in the thorax; if the mitral valves be diseased, there is more or less of a grating sensation communicated to the finger on applying it to the pulse at the wrist, and in either case the pulse intermits. The same occurs as to the grating sensation, when the heart is enlarged, and very considerable alteration of the lining membrane of the aorta above the semilunar valves, with dilatation of that tube, is found; atheromatous deposit under the lining membrane, with portions of bone being also found on dissection. In both cases, but more especially the latter, the patient is subject to very severe paroxysms of pain, lasting for the space of some minutes, hours, or even days, but alternating with intervals of perfect ease. The diagnosis of valvular disease is confessedly obscure. Even with the aid of auscultation, errors are frequently committed. If the preceding hints remove, or even materially diminish, the many sources of fallacy, they will be a valuable addition to our information.

"It is very remarkable, that disease of an artery may take place to a very great extent without dropsy occurring; while enlargement of the heart, unless death occur suddenly previously to the obstruction of the return of blood to the right side being considerable, is usually attended with dropsy during some portion of its progress. The effusion may indeed be got rid of by remedies, and the organs of the body, accustomed to the increased action, and greatly lowered in tone, still perform their functions, without a return of dropsy; but in such an instance frequently a few months only elapse before death, generally sudden, terminates the case." 13.

As the artery is rather the conductor, than the active agent in the circulation, it is less likely, à priori, that its morbid states should give rise to dropsy, than that either obstruction in the venous system, or alterations in the heart should do so. There is little, for example, in an aneurysm of the aorta, to occasion congestion in the limbs. But if the heart acts with too much or with too little force, or if impediments exist to the returning current of blood in the veins, congestions would appear to be the natural result. This seems the most reasonable explanation of the fact to which Dr. Seymour directs attention—a fact of great consequence in assisting our diagnosis between disease of the heart and of the arteries. The following case is well adapted to illustrate the point.

*Case.* "A patient in St. George's Hospital laboured under considerable pain in the region of the heart, without expectoration, and with only short dry cough, ability to fill his lungs fully on inspiration, and without pain. On inspecting the thorax, the heart was observed to beat very quickly, and somewhat strongly; its rapid motion being visible in the epigastrium, while the pulse in the brachial arteries was small, and there was no dropsy, neither had there been any effusion during the course of the malady. The extremely rapid and not weak action of the heart, combined with the weak pulse, appeared to me to denote that the heart was endeavouring to overcome some considerable obstruction to the circulation of the blood; produced by a tumour pressing upon the aorta, or by an aneurism. The case attracted considerable attention, and many gentlemen examined the chest very accuraetly; all believed the heart to be greatly enlarged, though much difference of opinion existed as to the presence of aneurism. My opinion was decided as to the absence of any except inconsiderable disease of the heart, because I had never seen a case with so much disturbance in the action of that viscus, arising from enlargement, without a greater or less degree of anasarca of the lower extremities.

About a year after the patient had been in the hospital, absorption of the ribs, at the posterior part of the thorax on the left side, took place; and a large aneurismal tumour appeared, which, after death, was found to have arisen in the descending aorta, and to bave caused absorption of the bodies of several of the dorsal vertebræ, but the heart was in an entirely natural condition." 15.

The dropsy which occurs in chlorosis appears to admit of a simple and consistent explanation, in the languor of the heart's action. Congestion of the venous system, and consequent serous effusion is the result, a result removed as well as its causes by the exhibition of steel, and the proper invigorating regimen. Profuse bleedings or hæmorrhage, act we may suppose, in a similar way.

Disease of the lungs, by offering an obstruction to the pulmonary circulation, is another cause of anasarca. If that is not so general, as in cases of diseased heart, it is because the obstruction to the pulmonary circulation is usually incomplete. The disease of the lungs, too, particularly if tubercular, is gradual in its progress, and the diminution of strength, and of the quantity of circulating fluids that has accompanied it, enables the parts, in some degree, to accommodate themselves to the change. On all these accounts the extent of the dropsical effusions is limited, and they consist in some œdema of the lower extremities, and in hydrothorax.

When the heart is enlarged with valvular disease, the anasarca is often great. The serous secretion relieves the pulmonary venous system, and preserves probably, for a time, the patient's life. When the mitral valves are extensively affected, and contraction of the left auriculo-ventricular opening has occurred, the right side of the heart becomes dilated and hypertrophied—the blood is driven with increased force *into* the lungs, and with increased difficulty *through* them—their vessels sooner or later give way—and what is absurdly called pulmonary apoplexy is the consequence.

" In many cases of extensive disease of the lungs, however, no dropsy is found. How is this to be explained? In such cases, expectoration takes place in a very copious degree; and this secretion constantly removed, and passing away from the mucous membrane, or from a false membrane in a vomica, affords the relief more safely to the congested circulation of the patient, than that which is effected by the effusion into the serous cavities, or the cellular membrane in other cases.*" 19.

Our author passes from the consideration of anasarca occasioned by disease of the heart or of the lungs, to that of anasarca resulting from the sudden application of cold to the surface of the body. When we remember the extreme vascularity of the cutis, and the quantity of sensible and insensible perspiration secreted by it, we cannot be surprised if a sudden check to the latter, should occasion increased quantities of secretion in other parts of the body. The mucous membrane is the internal integument, analogous in function, and almost similar in structure to the skin. The secretions of the latter being suddenly repressed, those of the former are augmented, and diarrhœa is the consequence. Diarrhœa is a common result of arrested perspiration—dropsy is an occasional one. In anasarca from this cause the vessels secrete lymph, the whole cellular membrane becomes distended, and the swelling is less elastic, more resisting, the skin more stretched and shining, than in the cases where a slow relief is afforded to the obstructed venous circulation. The action of the heart and arteries is increased in force and frequency; the secretion of urine is diminished, and high-coloured; the tongue is white; in fact, a feverish condition of the body is formed; blood drawn from the arm shews the buffy coat; there is intolerable thirst present; and the remedies, both indicated and effectual, are those which diminish the increased action of the heart and arteries, and control the secretion of coagulable lymph, viz. blood-letting and mercury.

Dr. Seymour alludes to the hypothesis that dropsical effusion is always an inflammatory process. That hypothesis is opposed not only to so many reasonable conclusions, but also to so many facts, that it does not appear necessary to engage in an elaborate examination of it. But it is certain that the limits between a passive exhalation of the serous portion of the blood, and a secretion of an inflammatory character are difficult if not impossible to be determined, and ordinary dropsy readily slides into the inflammatory form. The practical physician is aware of this, and carefully discriminates and closely watches the symptoms in each particular case.

The preceding general remarks on anasarca, introduce us to a more precise investigation of its several varieties. The second chapter treats of anasarca, dependent on rheumatic disease of the heart.

The subjects of this disease are generally young. A patient, four years

* " In cases also of solidified lung from tubercles, even where the expectoration is not considerable, diarrhœa or colliquative sweats being often present, relieve the obstruction."

of age, was admitted into St. George's Hospital on account of it. Dr. Seymour has seen it in patients nine years old; and the most severe case he ever witnessed terminated fatally, with great disorganization of the heart, at the age of thirteen.

"A patient labouring under anasarca from rheumatic disease of the heart, has swelling of the cellular membrane, having begun about the ankles: the face is also swelled, especially the eye-lids; there is an expression of anxiety, even greater than in diseases of the chest in general; the pulse is generally small and quick, sometimes intermitting, but very small compared with the action going on within the thorax; a tumultuous beating of the whole organ: this smallness of the pulse compared with the violence of the action in the heart itself, arises from the inordinate growth of the organ, without a corresponding enlargement of the vessels of the heart. In the most remarkable case of this kind I ever saw, the patient had laboured under the symptoms during four years, having commenced at nine years of age. The heart was enlarged to three or four times its natural size at his death, with repeated layers of lymph glueing together the pericardium: the aorta retaining the size of that vessel in a young boy of nine years. I have seen the same effect produced in adults, from contraction of the aorta above the semilunar valves. If the anasarca be very considerable, there is little pain felt in the chest, the obstructed circulation being relieved by the pouring out of the secretion from the minuter vessels. If the effusion be only commencing, there is almost always pain in the region of the heart, often pain in the course of the biceps muscle of the left arm, and cramps in the legs: the patient is unable to lie down, and the most easy position is bending forward over the back of a chair. There is also in the early stage a disposition to syncope.

If the effusion has taken place for some weeks, the pain and disturbance in the chest are less felt, and the patient attributes all his sufferings to the dropsy. The legs are tense; the face swelled, and of a pale bluish tint; great difficulty of breathing; the urine very scanty and high coloured, and where unaccompanied by disease of the kidney, does not coagulate by the usual method; the pulse quick and weak; the surface of the body cold; there is present a short dry cough without expectoration, or with a very slight frothy expectoration, occasionally tinged with blood." 26.

On questioning such a patient, we ascertain the previous occurrence of attacks of redness, swelling, and pains in the limbs. If he dies we find hypertrophy and dilatation of the heart, with adhesions (usually old, if there is dropsy) of the pericardium. Those adhesions are obviously the result of rheumatic inflammation. Dr. Seymour does not offer a minute history of this very interesting and important disorder, his object being rather to treat of its consequence—dropsy—than to enter on a circumstantial description of *its* causes.

He observes, with much truth, that in these cases, even when severe, the progress of the disorder is comparatively slow. The reasons that he adduces are, no doubt, correct. 1. The patients are generally young, and the growth of the chest allows it to accommodate itself to the enlargement of the heart;—2. Other viscera are usually sound, which is not the case in those forms of dropsy that arise from the abuse of stimulants.

The treatment of anasarca arising from rheumatic inflammation of the heart must vary with the period and state of the latter. If inflammation of the pericardium is still going on, the remedies must be antiphlogistic—cautious bleeding—calomel and opium—blisters. The diuretic which Dr.

Seymour prefers is nitre, exhibited in mint water twice daily, in doses of from ten to fifteen grains. He has not himself seen much benefit from colchicum; on the contrary, he has seen it occasion great depression of the vital power, and increased irregularity in the action of the heart.

But if the inflammatory symptoms have ceased—if the anasarca is considerable, and the skin pits deeply on pressure, the object is to procure a copious diuresis. In this case our author speaks highly of the effects of digitalis, a remedy first formally introduced by Dr. Withering, who remarked that it will be found most effectual in a lax state of the system, where the effusion is considerable, and the action of the heart and arteries feeble—a remark in which our author fully coincides with him. The form of digitalis preferred by Dr. S. is the infusion—the formula he offers is this:—

℞. Infusi Digitalis, ℥iv.

Liquor. Oxymur. Hydrarg. ℥ij.

Aq. Menth. Sativ. ℥viij.

Træ. Cantharidis, ♏xxx. M. Fiat haustus bis terve in die sumendus.

"The Tinct. Cantharidis, in itself an active diuretic, renders almost every other more effectual; but it is contra-indicated in aged persons, where disease of the urinary organs is present. The rapidity with which the above draught operates, is quite astonishing: I have seen as much as a gallon and a half of urine voided in one night, after the second day of its employment. From the presence of the oxymuriate, however, it will sometimes purge—an effect to be prevented; for this purpose, a pill, consisting of a grain of opium, should be given every night, at bed-time." 32.

In patients, whose circumstances relieve them from anxiety, exposure, and toil, the removal of the dropsy brings not only present relief, but a temporary though precarious respite from the disorder. But individuals in the lower classes of society quit the hospital only to be exposed to fresh exciting causes of the malady, and we cannot be surprised at the relapses which occur in them.

From anasarca following rheumatism, our author steps to anasarca occasioned by active enlargement of the heart. This is most commonly the effect of that unfortunate indulgence in spirit and porter-drinking, so prevalent in large cities. It occurs from twenty years of age to sixty, although it may arise at an earlier epoch. We lately saw a young gentleman, who died from dropsy dependent on hypertrophy and dilatation of the heart, before he had attained his twentieth year.

"The anasarca in this instance is enormous, the whole cellular membrane of the body being greatly distended; the integuments of the abdomen being increased to a size which appears at first sight impossible, without effusion into the abdominal cavity, and yet on striking the abdomen no fluctuation is perceived. In extreme cases, the distention is so great that the cutis splits into laminæ, giving the appearance of fine threads, dividing its texture transversely; at others, effusion takes place, in large blisters on the legs and extremities, which by bursting and discharging serum, for a time alleviate the distress. The pulse is generally strong and quick, the urine scanty and high coloured, and does not coagulate by heat, or the admixture of acid; the tongue is furred, the patient obliged to be raised in bed, and complaining of an insupportable oppression. The face is swollen, and often with a bluish tint, and there is constant thirst; the bowels are generally natural in their functions, and the appetite little deranged." 34.

When the anasarca has become excessive, the speedy relief of the distended integuments becomes absolutely indispensable. If the fluid is allowed to remain in such quantity in the cellular tissue, erysipelatous inflammation, first of the skin, then of the cellular membrane, will ensue. Formerly scarifications were made in the integuments, an effectual method of drawing off the water, but a very dangerous proceeding; for diffused cellular inflammation and gangrene very frequently followed it. Latterly punctures of the skin with a needle have been substituted for the scarifications. This is a comparatively safe operation. We are constantly in the habit of performing it, and it is seldom followed by any bad effects. The punctures should not be made in parts which will be subjected to pressure—they should only go into the cellular membrane, not through it—they should not be too numerous—nor should the operation be repeated at too short intervals. With these precautions we may count on seldom seeing mischief follow the use of the needle. A slight degree of diffuse inflammation of the skin and cellular membrane now and then occurs when punctures are made, but warm fomentations and bread poultices made with weak tepid goulard water usually disperse it. We lately, however, operated on a gentleman, labouring under dropsy from extensive disease of the heart, in whom the skin of both legs sloughed, after the acupuncturation. The patient died. Puncturing then is not devoid of danger, but it is infinitely less dangerous than scarifying.

What remedies must we employ, besides these merely mechanical means of easing the stretched skin? If the strength is yet unbroken, we may abstract some blood, and then employ a medicine of great power, in producing serous evacuations—the elaterium. Dr. Seymour begins with half a grain, and recommends the following formula.

> "℞ Elaterii*, gr. ½.
> Subm. Hydr.
> Pulv. Capsici, āā. gr. ij.
> Conf. Ros. Canin. q. s. ut ft. pilula, mane sumenda."

In private practice it is advisable to begin with a smaller dose than half a grain. We usually prescribe an eighth of a grain at starting, and we have seen such violent effects from a quarter of a grain, as obliged us to discontinue its use. The elaterium would appear to be of varying quality, and whenever it is procured from a fresh shop, or taken from a fresh supply, the physician should be informed of the circumstance. It has happened more

* "It must be observed, that the elaterium is most applicable in cases where the bodily powers are yet strong. Thus I was more successful in its use, in cases in the Seamen's Hospital, where the patients were hardened by exercise, and inured to exposure, than in St. George's Hospital, where many of the patients are from a class accustomed to long confinement and little exercise. Improperly administered, elaterium is apt to produce a diarrhœa, from which the patient never recovers.

Dr. Cullen used very successfully three grains of gamboge with sugar, every three hours. Such practice, though it produces frequent watery evacuations, is very inferior in power to the elaterium." 35.

than once that a furious purging has suddenly come on, and has been traced to the patient's having taken elaterium of a different quality from that which had been previously administered. The effects of this drug are frequently highly satisfactory, nay surprising, and it exerts almost a magical effect on the dropsical effusion. It produces watery motions, on which those effects depend. But it must be solicitously watched.

"When this remedy fails, or when it ceases to have the desired effect, I have succeeded in a manner which has greatly pleased and surprised me, in evacuating the fluid by large doses of cream of tartar. Small doses of this salt are among the most popular remedies for dropsy, increasing the flow of urine, whether as a beverage with lemon peel and ginger, under the name of Imperial, or with gin and sugar made into a kind of punch, whose diuretic properties are undoubted. But about seventy years since, Menghini, an Italian physician, recommended this medicine in far larger doses, as a deobstruent, and averred that he had cured many cases of dropsy by its exhibition.

He recommended an ounce to be taken every morning in water; the salt requiring about thirty parts of boiling water for its solution; and states that patients recovered with the use of no other remedy: he adds, that the urine let fall a sabulous deposit at the time." 38.

It is in this form of dropsy, that Dr. Seymour has seen the supertartrate of potass of most service. An ounce of it is given in solution every morning, or, if this acts too violently, the dose may be diminished to half an ounce. After the fourth day its beneficial influence generally commences.

Anasarca, from dilatation of the heart unattended with hypertrophy, is considered next. It is most frequently witnessed in the old, and is the result of intemperance, anxiety, watching, and long-continued profuse evacuations, all powerful causes of debility. Dr. Seymour assures us that it oftener exists in females than in males.

"The pulse is weak, often irregular; at least, even where there is volume, it is very compressible. The anasarca, which is very extensive, takes more the form of what has been termed leucophlegmasia: the face is swelled, and of a ghastly white appearance, while the integuments pit on the slightest pressure. The effusion is always preceded by difficulty of breathing, especially on any quick motion, by violent palpitation, and disposition to syncope. The difficulty of breathing is not, as in the former instance, much relieved at first by the effusion; for not unfrequently the cellular membrane connecting the lobules of the lungs is infiltrated also.

It is in this form of dropsy that sudden death frequently occurs. The patient may be apparently doing well, the swellings gradually receding, when a sudden return of effusion, either into the chest or pericardium, is followed by almost instant death.

This form also is often complicated with dilatation of the arch of the aorta, and deposition of atheromatous matter with spicula of bone under the lining membrane. In such cases there are not only all the evils arising from oppression and distention present, but also paroxysms of most severe pain, similar to what is experienced in ossification of the coronary arteries, and is termed angina pectoris." 41.

Perhaps exception may be taken to two statements in the preceding passage. The first is the observation that passive dilatation of the heart is often complicated with dilatation of the arch of the aorta, and with deposition of atheromatous matter and spicula of bone beneath the lining membrane. Setting aside our own observations, a large body of evidence has been adduced by Bouillaud and others, demonstrative of the co-existence of disease in the arterial tunics, including this affection of the arch of the aorta, with the forms of hypertrophy, rather than with simple dilatation of the heart. It is, indeed, most consistent with reasoning that it should be so; for whether the disease of the heart or of the artery arises first, it must naturally tend to induce the other. An inelastic artery demands more exertion from the heart, and a hypertrophic heart must necessarily tend to produce dilatation and disease of the artery.

The second remark to which we allude would tend, if we rightly understand it, to fasten the cause of angina pectoris on ossification of the coronary arteries—an opinion which is now pretty generally given up. Probably, our author does not intend to advocate it so exclusively as the apparent sense of the passage seems to do. It must be remembered, as we have already stated, that the references to the affections of the heart, and to their symptoms, are rather incidental than direct—and that our author must be held excused from discussing them at any length. This reflection will tend to account for the silence maintained on the subject of auscultation and percussion.

To return to the form of anasarca before us. It is in it, that digitalis is commonly most serviceable. It may be combined with squill and mercury, as in the pil. hydropica, (pil. hyd. gr. iij.; scillæ exsicc., pulv. fol. digitalis, āā. gr. i.) or in infusion with the liquor. oxymur. hydrarg. in mint water; and if the patient labour under no disease of the bladder or urethra, with the tincture of cantharides.

The mention of cantharides leads our author to pause, and to make a few remarks upon their powers.

*a.* Although Cullen, on the authority and in consequence of the experiments of Dr. Carmichael Smith, spoke slightingly of the cantharides as a diuretic, Dr. Wells is said to have found them of some service, and Sir Henry Halford informed Dr. Seymour, that he had observed them occasion a copious and a salutary flow of urine, in two severe cases of hydrocephalus. Hence our author was led to add this medicine to the infusion of diuretic vegetables; and the combination of tincture of cantharides with infusion of digitalis and oxymuriate of mercury has proved in his practice a powerful diuretic.

*b.* Dr. Seymour has derived considerable benefit from the cantharides, in the treatment of paraplegia. When it has done good in those troublesome, too often melancholy cases, it has displayed its diuretic properties. He relates two cases, and alludes to two more in confirmation of this statement.

We proceed with the drugs adapted for the treatment of the anasarca which forms the subject of the chapter.

"The nitrate of potass in solution, in the dose of fifteen grains; the acet. of potass, in the dose of from one to two drachms, either in its usual form, or made by neutralizing in solution the carbonate of potass with the vinegar of squills; may be given in a draught, with a pill, consisting of three grains of the blue pill, several times in the day; or a kind of punch may be given, made with cream of tartar instead of lemons, to every pint of which two ounces of gin may be added; all these are very effectual diuretic drinks. The latter is very convenient in hospital practice.

The spirit of nitric æther may be added, in the dose of a drachm, to a draught containing ten grains of nitre dissolved in mint-water; or it may be given as a beverage in the proportion of two drachms, or two drachms and a half of æther, in six ounces of water: in this latter form it is not disagreeable." 50.

Squill, combined with mercury, is adapted for those cases in which blood-letting is necessary, on account of its nauseating properties. When the stomach will not bear it, friction with it has been tried. The powder rubbed down with lard, in the proportion of half an ounce of the former to about an ounce of the latter, is the form proposed.* Our author says little in favour of the veratria ointment, or of the hydriodate of potass. He alludes to the old and bye-gone notion that drinking is injurious in dropsy, a notion embodied by Horace in the ode :—

> Crescit indulgens sibi dirus hydrops,
> Nec sitim pellit—

He passes in succession the infusion of the pyrola umbellata, an uncertain, an irritating, yet occasionally useful diuretic—the infusion or decoction of the broom tops—the garlic, which he has not seen of service—the infusion of the apocynum cannabinum or white hemp, of which he speaks disparagingly—and the application of cabbage-leaves to the legs, which have previously been gently rubbed with volatile liniment. As a resumé, he would place the particular diuretics in the following order, corresponding to their powers:—Infusion of Digitalis with tinct. cantharid. Nitrate of potass. Supertart. of potassæ with sp. juniper. c. Pil. hydrarg. pulv. dig. et scillæ exiscc.; in form of pill. Acet. et tinct. scillæ. Infus. pyrolæ umbellatæ. Infus. spartii. Sp. ætheris nitrici. Sp. armoraciæ comp.

In practice, diuretics may be simply arranged, as—those which exert some positive depressing influence—those which are little more than mere diuretics—and those which are stimulants too. It is the application of the one or of the other class to the features of the case and the condition of the patient, which constitutes judicious treatment. How frequently we see the most heterogeneous combinations—calomel, squills, digitalis, and gin, given at the same time, and apparently with the same view. It is true that in dropsy, as in other diseases, it is sometimes requisite to blow hot and cold, to support or even to stimulate at the very time that we employ special debilitating remedies. Such modes of treatment are particular, however, rather than general—the exception rather than the rule. And in this, as in other instances, the able physician is characterised by his application of those general principles both of reasoning and practice, which belong to science, not by an inveterate leaning to a faith in specifics, the sure evidence of empiricism and of art.

Our author lightly hints the necessity of those general measures—a mild diet—rest—pure air—passive exercise, and so forth, which will readily occur to the mind of the well-informed reader; and he deplores the ordinary fate of patients in the lower classes, whose intemperance commonly occasioned the disorder, and whose necessities, and too often whose resumption of intemperance, combine to render relief transient, and a fatal termination speedy.

---

* One drachm of this ointment may be rubbed in three times daily.

He makes a concluding observation, the justice of which will be acknowledged by all who are practically conversant with dropsy.

"It sometimes happens that after a long and very unsatisfactory course of treatment, with little effect in reducing the swellings, and thus relieving the oppression of the patient, the fluid, after the administration of some trifling remedy, begins suddenly to subside, with a rapidity equally astonishing and cheering to the patient: his attention being always fixed upon the dropsy, the symptom of the disease, he feels delighted at getting rid of what he considers to be the whole malady.

Such subsidence of the effusion in dropsy, unpreceded by a gradually increased flow of urine, or by the use of remedies calculated to produce evacuations, and which by being administered during a long period, hinder, at least for a time, the re-collection of the fluid, is a very fatal symptom; the vital powers are no longer sufficiently strong to afford relief to the obstructed circulation, secretion from the smaller and more minute vessels no longer relieves the labouring heart. In several cases which have fallen under my observation, the patient has never rallied after this sudden subsidence of the effusion beyond a month: but I have heard of life being prolonged somewhat longer. A case of this kind is to be found in the experience of the late Dr. Parry, of Bath." 57.

We have arrived at the fourth chapter, and we leave behind us the organic diseases of the heart. The form of anasarca, which is now to occupy us, and to which this chapter is appropriated, is independent of any lesion of an internal organ.

1. The sudden impression of cold and wet on the surface of the body, though it usually gives birth to other mischiefs, will at times occasion anasarca. The swelling is hard and tense, the pulse hard, the urine very scanty and incoagulable by heat or acids, the heart beats without symptoms of organic disease, though its action is increased, the bowels are costive. There is in this state a general feverish excitement; the vessels of the skin, over the whole surface of the body, secrete serum, and the mucous membrane is deprived of the natural secretion which these vessels pour out in a state of health. The sudden sequence of the anasarca to the application of cold, the probably previous good health of the patient, and the absence of the symptoms of any organic cause of the disease, are the distinctive marks of this form of dropsy.

The treatment is obvious, and generally successful. Bleeding (the blood is almost always cupped and buffed)—calomel and opium—saline purgatives—protection from the influence of cold, are its main items. The patient should not venture out too early.

2. It is well known that scarlet fever is occasionally followed by anasarca. Its pathology is not well understood, but it seems to be less frequent and less fatal than formerly. Its origin has been attributed to cold—to weakness—and Dr. Seymour leans to its being the result of deficient purging, for he has seldom if ever seen it, where saline remedies were freely used, especially towards the decline of the disease. It is not improbable that diarrhœa, natural or artificial, may tend to prevent the occurrence of anasarca, the efficient cause of which is, perhaps, to be sought in the excessive vascular action of the skin in scarlet fever, and in the necessary congestion of its vessels, which traverse the subcutaneous cellular membrane. Yet the liability to anasarca does not seem to be in the ratio of the severity of the scarlet fever, a slight attack of which will sometimes give rise to it.

"Anasarca, after scarlatina, begins generally from the fourteenth to the eighteenth day after the commencement of the disease—sometimes gradually, sometimes within the space of a very few hours; the patient is enormously swollen, so that the eyes are closed; the countenance white, shining, and swollen to an enormous extent; the secretion of urine of a deep colour, and in very small quantity. In many instances the urine is mixed with blood, and in such cases coagulates. (See a paper by Dr. Wells, in the first vol. of the Transactions of a Society for the Improvement of Medical and Chirurgical Knowledge.) There is thirst, a furred tongue, and even, on the onset, heat of skin; sometimes, but rarely, difficulty of breathing and headache: the integuments of the abdomen as well as the scrotum, and the rest of the cellular membrane of the body, are anasarcous in the highest degree." 66.

The treatment should be antiphlogistic, at least if the previous fever has been slight, and if the strength of the patient is unbroken. Bloodletting may pave the way for smart purging, with calomel, and a diuretic drink, made, perhaps, with the spiritus ætheris nitrici. If, however, the patient is debilitated, bleeding and active purging would obviously be improper, and we must chiefly lean on diuretics. The combination of the infusion of digitalis with the infusion of gentian, to which may be added the liquor potassæ—or the decoction of the pyrola umbellata, with the tinctura lyttæ, have been highly serviceable. In no case has our author seen tonics alone successful. He hints that the vapour-bath may be useful—but bloodletting must have been premised.

Anasarca, however, is not the only form of dropsy witnessed after scarlet fever—nor is it the most formidable. Ascites, hydrothorax, and hydrocephalus may occur, and although they are of course more tractable than when created by organic lesions, they must be looked on as more serious than anasarca. Dr. Seymour relates an interesting case, in which effusion would seem to have taken place within the cranium. The interest is physiological as well as pathological.

*Case.* A married woman, aged 36, was admitted some years ago into St. George's Hospital, under the care of Dr. Seymour, with a slight attack of scarlatina: the efflorescence was bright, continued three days; the throat was slightly ulcerated; and the cuticle desquamated as usual. During the efflorescence she was sponged with cold water and vinegar; the bowels were freely opened; first, fomenting gargles, and subsequently astringent ones, were used to the throat, and the case proceeded to convalescence readily. At the expiration of ten days she appeared to be quite well, and remained only in the hospital as a precaution against taking cold. About the fourteenth day from the commencement of the disease, she was suddenly attacked with pain in the head, loss of speech, followed by paralysis of the sphincters, dilatation of the pupil of the eye; all which symptoms were formed in the course of a day and night. The pulse was quick and small, and there could be no doubt of effusion having taken place into the head. She was ordered to be bled to sixteen ounces; two grains of calomel were put upon the tongue every three hours; and a blister applied to the top of the head, and kept open with mercurial ointment; half an ounce of the infusion of digitalis being put down the throat with a spoon, the intermediate four hours.

She gradually recovered. But the integrity of the mind was very slowly re-established. She understood all that was said to her, but could not remember the words to express her answer. Thus, when asked what o'clock it was, she explained herself by signs, not being able to remember the words: subsequently she used words, single, and unconnected by articles, so as to express her meaning, but not in a sentence. By degrees this power of expression returned; and at the end of a month she was quite well. Two years afterwards, she was seen by Dr. Seymour and remained in perfect health. It is difficult to reconcile cases like these (and there have been many) with the opinion, that different portions of the brain do *not* perform different intellectual functions. What we call the intellect is indeed an aggregate of many faculties. Metaphysical analysis seems capable of resolving and of isolating them. Whether that analysis be accurate or not, such cases as the preceding conclusively prove that a coarse organic lesion, a tea-spoonful of water, a particle of lymph, or a spiculum of bone, will play the metaphysician's part, and single out some particular mental quality for impairment or destruction. In this instance, there was a loss of the memory of words.

At times scarlet fever is accompanied or followed by a very decided, and a very dangerous inflammatory effusion in the chest. Empyema is the consequence. There have been several cases of this kind in St. George's Hospital.

3. Anasarca occurs in cases of chlorosis, or it may ensue after great loss of blood. Its cause is of a similar character in both cases. The heart having too little, or too diluted blood to circulate, would appear to make up in rapidity of action what it wants in force. Dr. Seymour conjectures that the blood shews a disposition to stagnate in the right side of the organ. Perhaps this may be questioned. But whether the supposition is correct or not; whether the immediate cause is or is not congestion in some portion of the venous system, it is certain that the serous part of the blood is apt to be separated, and that partial or general anasarca ensues. The ancles and legs swell, the face is puffy and white, the pulse in slight cases is languid, in severe ones rapid and weak, there is palpitation, headache, hurried or difficult respiration—symptoms, in short, very characteristic and very familiar.

The treatment in such a case must be gently tonic; yet the physician must feel his way, for tonics may not be borne at first. We need not mention the principles that should regulate our exhibition of the fetid gums, ammonia, steel, nor need we dwell on the necessity for pure air, appropriate exercise, occupation or amusement of the mind, those general measures which assist the operation of medicines so much, and without which medicines are comparatively powerless.

The fifth chapter is dedicated to the examination of a disease, which may be said to have appeared within these few years on the medical horizon, and for our knowledge of which we are principally indebted to the diligence of Dr. Bright. It is anasarca arising from disease of the kidney.

The distinctive marks of this interesting and formidable malady may be readily enumerated. The anasarca is rather seen in the lower than in the upper portions of the body. Yet the face is usually puffy, and the skin is singularly pale and dry. The absence of decisive symptoms (and here auscultation is peculiarly valuable) of affection of the heart, shews that that organ is not the cause of the dropsy. And the coagulability of the urine, on the application of acids and of heat, is the evidence of alteration of the kidney. The specific gravity of the urine is always diminished, and there is frequently a deficiency of urea in it.

The quantity of the urine varies. It is sometimes scanty and high coloured; but in the worst cases the urine has been found to be pale and abundant, while the anasarca was general and to a great extent. In such cases the disease is very fatal; and the condition of the kidney, its extensive disorganization and destruction visible after death, explains the reason why anasarca, without any diminution of the secretion of the urine, was always looked upon as a condition of disease unusually hopeless.

In the slight cases, in which the coagulability of the urine is least decided, the kidney may be simply congested or engorged. In the severer cases, or, in other words, when the disease has made more progress, the quantity of albumen in the urine is augmented, and the kidney has assumed the granulated state so well described by Dr. Bright. This concurrence of symptoms and lesion is now established as a general fact, by numerous and accurate observers.

It not unfrequently occurs that we discover after death, disease of the liver and the heart, in addition to disorganization of the kidney. It is well known that Dr. Bright has ingeniously argued that the kidney was the organ primarily affected, and that the disease of the heart resulted from it. This is perhaps the weakest point in the observations of that able pathologist, and we quite concur with Dr. Seymour in the following remarks upon the subject.

"Where disease of the liver, heart, and kidneys, exist in the same patients, Dr. Bright appears to believe that disease of the kidneys is the primary disease, and the disorganization of the other important structures secondary. It is more clear to me, that the same cause, the abuse of ardent spirits, or long-continued and repeated exposure to cold, which has produced disease in one viscus, has also affected the others. We know that disease of the liver and of the heart proceed from intemperance; and instances are not wanting of disease of the kidney producing anasarca arising from this cause, and existing alone. One of the worst cases I ever saw, of anasarca with coagulable urine, was in a youth of nineteen, who had been addicted to the immoderate use of spirits, rum principally, and no other viscus was diseased except the kidneys. Again, how often do we not find, after hard drinking, enlargement of the heart and liver accompanied by anasarca and ascites, while the kidneys remain perfectly sound?" 76.

A very good proof of the exactness conferred on medicine by morbid anatomy is presented to us by this very affection. It is only within these last few years that we have learnt the dependence of anasarca on renal disease, and we find that it exists in a very large proportion of cases. Dr. Seymour makes the following rough calculation of the relative number received into St. George's Hospital.

"About half the cases of dropsy admitted into St. George's Hospital, in my experience, have coagulable urine: and in about one-third of such of these cases as have proved fatal, the kidney was the only viscus diseased: the other two-thirds had disease of the heart and liver in addition to granulation of the kidneys. Of those cases of anasarca without coagulable urine which proved fatal, the heart was invariably greatly enlarged; and these were often complicated with ascites from disease of the liver." 76.

There are some circumstances connected with coagulability of the urine and organic alterations of the kidney, which may not improperly be glanced at here. We shall merely hint at them, because their consideration would occupy us long, and withdraw us from the pursuit of the history of dropsy.

1. The urine, after scarlatina, may contain an admixture of blood, and be, of course, coagulable. And in dropsies from cold, accompanied with an inflammatory condition of the system, coagulability may also be present, and is removeable by antiphlogistic treatment. In these cases we must suppose that the functions of the kidney only are disturbed—a view borne out by numerous facts which prove that an albuminous state of the urine is not necessarily dependent on organic lesion.

2. There is no doubt that several organic lesions of the kidney will give rise to coagulable urine. They would appear, however, to have this in common, that they are attended with congestion or a state of chronic inflammation of the organ. It is difficult to say, why in one case dropsy is present, whilst in another it is not—and it is still more difficult to understand, why furious symptoms of irritation of the bladder should exist in one instance, and be totally absent in another, where the organic lesion is apparently of a similar description. We may fairly conclude that the diseases of the kidney have not yet received all the elucidation they are capable of, certainly not all that is desirable.

The study of morbid anatomy is apt to lead those who pursue it very closely, into an inert method of practice. Constantly witnessing extreme organic lesions, they insensibly contract the notion that it is idle attempting to remove such structural alterations by remedies; and they forget that what they witness is the termination of actions that may in many cases be controlled. It is certain that the best pathologists are not unfrequently indifferent practitioners, and whilst their diagnoses have all the accuracy which science can confer, they do less for the cure of the disease they have discriminated, than comparatively ignorant but decided men who confidently trust to their empirical experience. It is the combination of the knowledge of morbid anatomy with the numerous resources of practical experience, that constitutes the accomplished physician or surgeon.

If we have determined that anasarca is frequently the effect of organic alteration of the kidney, we must not therefore sit down in despair and abandon the patient to his fate. Those organic alterations though irremediable when far advanced, present no doubt remediable stages, and it is our business to ascertain by experiment what can be done for their mitigation or removal. Fortunately the intimate sympathy which exists between the kidneys and the skin, opens to us a mode of acting on the latter, a mode which has been seized by observant practical physicians. Dr. Osborne, in a work on dropsy, has particularly drawn attention to the necessity and the advantage of inducing perspiration, when that is possible, in the form of dropsy before us. The following are the observations of our author, which are strongly in support of Dr. Osborne's views, and calculated to encourage us in our efforts to relieve our patients.

"Dr. Wells cured three cases out of five, of anasarca with coagulable urine, with cantharides; and Dr. Mead recommends them in lepra, from their diuretic property. Dr. Bright states that he has succeeded with the cream of tartar in several instances, and I have found this medicine far more useful than any other in dropsy from disease of the kidney; but looking to the functions of the skin, I have employed also the vapour bath, using this remedy on alternate days. The perspiration excited by the vapour bath is excessive, and its use should be graduated to the patient's strength. I have found this remedy far superior to any internal one known to produce diaphoresis. Where there is pain in the lumbar region, as not unfrequently occurs in an aggravated form of the disease, cupping is very useful, and every intercurrent inflammatory disease I have been in the habit of treating exactly as if no dropsy had existed, according to the symptoms and locality—blisters only being excepted, from their being often followed by erysipelas, and even sloughing." 78.

A case is related which exhibits the good effects of this rational system of management. It is not necessary to introduce it here. But before we dismiss the subject, we may allude to the frequency with which inflammatory attacks of other organs occur in patients with diseased kidneys. This is a fact which has been pointed out by Dr. Bright, who has also dwelt on the liability of such patients to cerebral oppression.

### ASCITES.

The second part of Dr. Seymour's work takes up the consideration of ascites, or dropsy of the abdomen. This may depend, as we have seen anasarca do, on disease of the heart or of the kidneys, or rather it may exist with anasarca in those cases. In them, however, it is usually both inferior in degree and in importance. Where the kidneys only are diseased, our author thinks that it is not generally perceived; and when the heart only is affected, it is certainly subordinate to the infiltration of the cellular membrane. The explanation of this circumstance which he offers, is probably the just one. The circulation in the abdomen is peculiar: the greater quantity is expended in forming the important secretion of bile from the vena portæ, and the remainder only is returned by the hepatic veins to the right side of the heart. The obstruction therefore to the heart's action, arising from enlargement or disproportionate dilatation of the cavities of the organ, will affect the venous system in the abdomen far less than throughout the whole body: hence it is that anasarca is the most common form of dropsy attendant on disease of the heart, and where this important viscus is alone diseased, ascites scarcely appears.

The principal cause of ascites is disease of the liver. There have been many opinions on the manner in which hepatic disease gives rise to it, and even now the minds of pathologists would appear to be far from made up with respect to it.

The old, and perhaps the common idea, attributes ascites to the bulk of the liver, which, pressing on the vena portæ, produces congestion in the capillary veins from which it springs, and, as a consequence of that congestion, serous exhalation. But this notion seems overthrown at once by the fact, that simply enlarged liver is found to be rarely a cause of ascites, and that when the organ is much increased in bulk by depositions of encephaloid tubercles in its structure, ascites is not necessarily present.

"The most frequent appearance of disease of the liver, and that which is most generally connected with ascites, is characterized by diminution of the bulk of the viscus: the liver is about one-half its natural size, very hard, and with the sharp edge, so characteristic of it in a state of health, blunt and rounded.

In post-mortem examinations, instead of finding it protruding into the belly, we seek for it under the ribs in the right hypochondrium, and find it there drawn up, as it would seem, from its diminished size. The peritoneal coat is in such case most frequently found thickened: this not unusually takes place in parts, and this partial thickening gives to the viscus the appearance of being puckered, not very unlike the lobulated structure of the calf's kidney. On cutting into these portions, the viscus itself does not present corresponding depressions, but the appearance arises from the membrane having been thickened in parts. Occasionally the whole peritoneal coat is thickened, of a milky white colour, and the viscus beneath contracted, extremely hard to the knife, and presenting a round almost globular mass when removed from the body." 85.

The thickening of the peritoneal coat has led some authors to suppose that inflammation of it alone would be sufficient to occasion the disease. The cause appears, on a close examination, disproportionate to the effect, and it seems difficult to admit the supposition, that inflammation of the peritoneal investment of the liver is able to produce what much more extensive peritoneal inflammation does not. The explanation offered by our author is much more consistent with our general pathological knowledge. He assumes that the result of the change in the viscus alluded to is—uniform pressure on and obstruction of the minute and secreting branches of the vena portæ. The consequences of such obstruction are obvious.

To support this view he alludes to another case of ascites, in which the secreting structure of the liver is nearly obstructed by the deposition of a reddish-white substance, by some believed to be lymph, and the effect of inflammation, by others supposed to be cholesterine; but in either case, on a section of the liver, the whole seems to be made up of rounded masses, which, in some instances, can be separated from the cellular membrane which connects them, though the change is uniform throughout the whole mass. In many such cases the peritoneal coat is quite clear and transparent, proving that causes exist, independent of its thickening, to produce such an obstruction to the progress of the blood, as to act as the proximate cause of dropsy.

A difficulty indeed occurs, and it may naturally be demanded, how, if this physical explanation be acceded to, shall we explain the absence of ascites, in cases where the structure of the liver is quite altered—where its parenchyma is converted into a fatty mass, or of a very pale yellow colour, or almost superseded by encephaloid tubercles? Our author anticipates the question, and offers the following reply.

"That such cases have occurred there can be no doubt; and it appears to me to be accounted for by the alteration in the circulating fluid itself: there is a degeneration of the whole body, the arteries do not secrete fluid, in order to keep up the balance of the circulation, impeded by the disease of the viscus; but both solids and fluids undergo a degree of degeneration, and the whole system tends to decay. The appearance of persons labouring under this form of disease confirms this idea; the countenance is of a waxy yellow colour; no red blood appears circulating on the surface; the principal ailment complained of is excessive weakness, sinking, often without any local pain; the pulse is weak, and about a hundred in a minute; the patient is emaciated, and the muscles have lost their firmness and colour; in blood drawn from the arm, the red particles are scanty, and the fibrin scarcely adheres; a weakened state of the nutritive fluid, adapted to the weakened state of parts which act in the function of secretion, and both tending to the undoubted death of the patient." 87.

We fear that this reply will be deemed inconclusive. Granting the general decay insisted on, that does not satisfactorily explain why a physical cause should not be succeeded by a physical effect. The debility which exists in these cases, would appear, from what we know of dropsy, to be rather favourable than otherwise to its occurrence, and that very debility and anæmia have been seen, in chlorosis and after exhausting hæmorrhages, to give rise to anasarca. It must be owned that some obscurity surrounds the immediate cause of dropsy. In some cases physical obstructions to the course of venous blood, or obvious congestions in the venous system, afford a ready and a simple explanation of the serous effusions that ensue. But in others, we look in vain for such satisfactory causation. It is not for example clear, why, on the principles alluded to, disease of the kidney should give rise to dropsy—why hydroceles should occur, as they sometimes do, in young and comparatively healthy persons. It is probable that the condition of the blood itself, as well as of the solids, exerts an influence which is far from understood, in the production of some of these effusions. Be that as it may, we leave the consideration of this question to proceed with the history of ascites. Let us again observe, that, according to our author, its most frequent, most obvious, and most indomitable form arises from a hard, contracted state of the liver, with a thickened peritoneal coat—while a second kind results from an universal alteration of its secreting structure, by the deposition of a reddish-white matter, supposed to be coagulable lymph by some, cholesterine by others, and unaccompanied by any change in the peritoneal investment.* The picture of an ascitic patient drawn by Dr. Seymour will be recognized as a faithful one.

" The external appearance of a person labouring under ascites from either of these causes is in striking contrast with those cases of anasarca which arise from disease of the heart. When some induration of the liver accompanies disease of the heart, both ascites and anasarca are present, but the abdominal effusion is small in quantity, compared with that which takes place in the cases I am attempting to describe: in the former, the increase of bulk is principally owing to infiltration of the cellular membrane connecting the muscles of the abdomen; in the second, these muscles are wasted, the fat absorbed, the distended abdomen is tense in every part, the swollen veins give to the whole a bluish appearance, while the hands and arms are wasted, the features drawn in and haggard, presenting the appearance of what has been termed 'facies hippocratica;' the legs are also often wasted, and indeed always, early in the disease, but at length the pressure of the immense body of fluid in the pelvis prevents the return of blood in the iliac veins, and the lower extremities become anasarcous. The pulse is quick and feeble; the tongue red, and rough with prominent papillæ, and sometimes aphthous; the urine is very scanty, and deposits a pink sediment, like the finest rouge; the thirst is insupportable; and the alvine excretions scanty and ill-coloured, the bowels being sometimes difficult to move, at others affected with diarrhœa." 89.

Unfortunately the treatment of such a case may be speedily dispatched. What can we reasonably expect to do? In a recent case, mercurials, moderate purging with senna and crystals of tartar, diuretic drinks, especially a

* We may observe, by the way, that it is doubtful whether the thickening, said to occur at times in the peritoneal coat, has really that site. It is more likely to exist in the cellular capsule, beneath the peritoneum. Such appeared to be the case in some dissections which we made, but we have not examined the matter narrowly.

solution of nitre, perhaps, for those who have faith in its *mild* virtues, the dandelion, are remedies appropriate, we might wish to add, successful. When the disease is far advanced, the judicious physician thinks of little more than relieving suffering and supporting strength. Mild tonics, especially a weak acidulated solution of quinine, moderate purgation, and when the quantity of fluid demands it, the operation of tapping, almost compose our means of treatment.

The period at which tapping is necessary, has occasioned some difference of opinion in the profession. While some have recommended it as soon as the fluid has become very abundant, others have advised a fair trial of medicines first. On the whole, there can be little doubt of the propriety of the latter opinion, especially in private practice, where both patient and physician are anxious to avoid an operation.

The character of the fluid abstracted is not in all cases the same. Where the liver has been contracted during a long period, has a puckered peritoneal coat, or that membrane is thickened like semi-transparent horn, its sharp edge having entirely disappeared, or where the secreting vessels are uniformly obstructed, the fluid drawn off is quite limpid, and enormous in quantity. Often the fluid is of a pale yellow colour, more or less tinged with bile, and it soon coagulates on standing. In young and strumous patients, or at least chiefly in them, in whom the collection of fluid is not large, it is of the colour and consistence of whey, with shreds of lymph floating in it; and is undoubtedly the product of inflammatory action, modified by the constitutional disease we have mentioned, for where acute inflammation of the peritoneum terminates in similar effusion, it uniformly proves fatal: it is the product, therefore, of a slower inflammatory action than in acute cases.

"If clear serum, but deeply tinged with blood, follow the introduction of the trochar, we may be certain that a malignant growth of considerable size, (so large as to press upon the blood-vessels) often attached to the liver or ovarium, is the cause of the peritoneal dropsy. I am not to be understood to say, that when a large morbid growth in the abdomen, of the kind termed fungoid or malignant, proves fatal, accompanied by dropsy, that the serum is always bloody; but that this bloody serum, when it does occur, always depends on such morbid growth. In many cases in the hospital I have seen this remark verified, so that I believe it to be strictly correct." 93.

If the sudden disappearance of the fluid in anasarca was dangerous, it is also a harbinger of evil in ascites. In the latter, however, it is preceded by frequent and almost uncontrollable vomiting, or, if the water has been drawn off by tapping, and does not reaccumulate, violent pains in the bowels occur, accompanied with diarrhœa difficult to be restrained. Dr. Seymour has found in such cases, after death, the intestines of a deep leaden blue colour throughout their whole extent, and the omentum of the same colour. The peritoneal investment of the intestines is thickened and opaque, and sometimes shreds of lymph, not of very recent formation, adhere to the convolutions. There can, we presume, be little question that this blue colour is the result of congestion, insensibly gliding into low and chronic inflammation: the thickening and the lymph are sufficient evidence of that.

In some cases, and this is especially the result of repeated attacks of ague, or of residence in a pestiferous district, the liver and spleen become greatly enlarged. If the former is simply enlarged, without hardness, effusion into the abdomen may not follow—if enlarged and indurated, ascites, less considerable and less intractable than when dependent on a small hard liver, is the consequence. The familiar explanation of the enlargement of the liver and spleen after agues, is no doubt the true one. The reiterated congestions in the abdominal viscera occasioned by the cold fit, terminate in the morbid growth of those two organs, in which, from their anatomical structure, such congestion must be felt most sensibly.

"Fortunately, except under very aggravated circumstances, the effusion and its cause are removed, and the patient permanently cured, unless constantly exposed to the miasmata. Mercurial frictions have often a most extraordinary effect in this disease, reducing the swellings, and from their diuretic power carrying off the effused fluid at the same time. To these saline purgatives should be added; and it is remarkable that mineral waters possessing purgative qualities are often found in the neighbourhood of marshy districts, affording the ready means of relieving the diseases engendered by a sojourn in these unhealthy regions. The waters of St. Filippo, in Tuscany, are annually resorted to for this purpose: they resemble in some measure the waters of Harrowgate, often drunk in our country, to relieve the chronic diseases of the liver, contracted during a residence in India. The waters also of Monte Catini, near Pisa, are celebrated also in that country, whose marshy districts abound with ague, for the cure of visceral obstructions.

The practice also of letting fall a column of hot water on the affected part, under the name of *douche*, affords a very effectual method of reducing the swellings of the viscera. This practice in France and Italy, in such cases, is almost universal.

Modern discoveries also have afforded a most powerful agent in reducing these swellings, when of long standing, in the hydriodate of potass. Two or three grains of this salt, taken, dissolved in two or three ounces of water, twice daily, will sometimes succeed when other remedies fail: under its use the patient's strength and appetite will return, while the dropsical effusion subsides gradually, with an increased and limpid flow of urine. Of course, if the enlarged viscera be painful to the touch, it will be advisable to apply leeches repeatedly, before having recourse either to mercurial friction or the use of the hydriodate of potass. Where mineral waters cannot be obtained, the best saline medicines are the tartrate or supertartrate of potass, or the Rochelle salt (tart. pot. et sodæ), in the dose of from half an ounce to six drachms, every morning, dissolved in half a pint of water." 99.

There is reason to believe that iodine is applicable to the treatment of more cases of ascites, than that dependent on enlarged liver. We have seen it of great service, where there was no reason to believe that enlargement of the organ existed, in any material degree. The combination of the tincture of iodine with the hydriodate, appears to augment the powers of the remedy. Iodine, however, must be given cautiously, and never when there is a tendency to inflammatory action. Its primary effects are frequently stimulating, or, at least, it seems to induce, in some cases, inflammatory determinations. On the other hand, if injudiciously exhibited, it may suddenly depress, and occasion low fever from its effects upon the gastro-intestinal mucous membrane.

We must soon part from our able author, for we have arrived at the sixth chapter, the last of the original portion of the book. It treats of ascites from disease of the peritoneum, that is, from the tuberculated condition of it, described so well by Dr. Baron, of Cheltenham. The disease in question consists, in its early stages, of a pearly thickening of the peritoneum, the membrane being pushed up by numerous eminences, or tubercles deposited on the cellular side of the serous membrane; in more chronic cases, large masses of thick cheesy-looking matter are deposited on the cellular side of the omentum, sometimes distending it, like a bag, into an unequal globular tumor; at others, into a thick flat mass, compressing the intestines like a pad.

About thirteen years ago, Dr. Cholmeley, of Guy's Hospital, drew our author's attention to the case of a patient who vomited a peculiar deep leek-green coloured fluid, precisely resembling in tint the beautiful fluor spar, found at Alston, in Cumberland. Dr. Cholmeley stated that the patient in question laboured under the tuberculated state of the peritoneum, for whenever he had seen this vomiting, he had found on dissection that disease. So it turned out, and Dr. Seymour has since had many opportunities of confirming Dr. Cholmeley's remark. Yet he has not observed this peculiar vomiting when ascites has accompanied the tubercular accretions, probably (so he thinks) because the evacuation by vomiting prevents the serous effusion.

To return, then, to the ascites. It is always more scanty than when caused by hepatic disease. Two gallons would appear to be the utmost extent, whilst in some cases only a few pints are present. The fluid is more or less whey-coloured, and often mixed with shreds of lymph, a plain proof of its inflammatory character. It more frequently accompanies the universal thickening of the peritoneal covering of the bowels, than accretion of the omentum.

It is more common in young than in old persons, in females than in males. It occurs in strumous habits, and appears to arise from inflammation, modified by that constitutional state.

The pulse in such cases is weak and quick; the features pinched and sharp, and the tongue red and glazed, as if a hot iron had touched it; sometimes of still deeper red colour, with patches of aphthæ. There is always a sense of weight and distention in the abdomen, even where no ascites is present.

"I have observed that where effusion takes place, it often arises early in the disease; whereas, where the disease is fully established, and the prolongations of the serous membranes are formed into hard masses, it is more frequently absent, while peculiar and fatal vomiting relieves the obstructed circulation; and hence it is, I think, that I have seen several young patients rally, where the dropsy appeared early from this organic affection." 104.

If the treatment is too often unsuccessful, happily the disease is sufficiently uncommon to diminish our regret at the comparative impotence of medicine. Mercury is rarely advantageous—powerful purging is obviously unsafe—tapping is injurious, as a slight consideration of the cause of the effusion might lead us to suppose.

"If, when the physician is consulted, the pain on pressing the abdomen be severe, one bleeding from the arm will be necessary, or if the patient be weakly, a dozen leeches may be applied to the abdomen: further than this, antiphlogistic practice appears to be hurtful, and in many cases the practitioner is not consulted until the results of slow inflammation have been established; thus rendering depletion improper, as weakening the forces of the patient, without controlling the action which first produced the disease.

Frictions with the liniment hydrarg., morning and evening, are often attended with benefit, but it is on the use of the hydriodate of potass that I have been accustomed to rely, from finding the general health recover under its influence, and the effusion, hardness, and tightness of the abdomen, disappear in a remarkable manner. Two grains of this salt, given in a draught of equal parts of cinnamon water and common water, with a little syrup, may be administered for several weeks, twice in the day, with no other visible effect than the amendment of the patient's health, or if any visible effect is produced, it is slight diuresis: even during its employment, a few leeches may occasionally be applied with advantage where there is any pain on pressure: if restlessness and sleeplessness be present, five grains of the ext. conii may be given with each draught, in the form of pill." 106.

The aperient which our author has found to agree best has been the phosphate of soda, from half an ounce to six drachms of which may be given daily in a pint of beef tea. It is not disagreeable, and is borne well by the stomach, irritable as that commonly is.

Ascites is sometimes the consequence of other diseases, of a tumor for instance of the ovary, or of enlargement of it, capable of compressing the veins which form the vena portæ. In such a case the introduction of the trocar first draws off clear serum from the peritoneal sac, then ropy muco-albuminous fluid from the ovarian cyst. In these cases the recumbent posture and diuretic remedies will often relieve the ascites remarkably, and after a time the tumor may possibly shift its place, the injurious pressure may be removed, and the peritoneal effusion may recur no more.

It has been supposed that rupture of the thoracic duct may cause ascites—an improbable conjecture unsupported by actual examination. Nor is simple enlargement of the mesenteric glands succeeded by ascites. Marasmus is its usual effect, though ascites may ensue if the glands press upon the vena portæ, or if, as sometimes happens, the growth is accompanied with chronic scrophulous inflammation of the peritoneum.

Ascites is not so frequent a sequela of eruptive diseases as anasarca is. When it does occur, antiphlogistic remedies and saline purgatives are probably best adapted for its early stage, and in its latter ones, it has been cured by the infusion of the pyrola umbellata, whilst the spartium scoparium, and the infusion of the root of the ononis spinosa have been recommended for it.

This concludes Dr. Seymour's own portion of the volume. The remainder is a translation of Geromini's work. As that is rather a history of dropsy than a practical account of the disorder, it is not adapted for our present purpose. Yet there is much in it that deserves perusal, and we shall take care, at an early opportunity, to lay a sketch of it before our readers. It seems scarcely necessary to pronounce a formal opinion on the matter contributed by Dr. Seymour. We have presented it in a complete and close analysis, and we think there can be no doubt that *that* amply bears out the favourable sentiments we expressed at the commencement of our notice of it. We need not add that the author is a physician of high practical attainments, who has laid before the public, in an unaffected manner, the results of accurate observation and of considerable experience.

